# Clinical significance of apelin in the treatment of type 2 diabetic peripheral neuropathy

**DOI:** 10.1097/MD.0000000000025710

**Published:** 2021-04-30

**Authors:** Hua Xu, Qi Wang, Qian Wang, Xuan Qiang Che, Xue Liu, Shumiao Zhao, Shitao Wang

**Affiliations:** a Department of Endocrinology; b Department of Pharmacy, The Fifth People's Hospital of Jinan; c Department of Ultrasound, Shandong Provincial Hospital, Jinan, Shandong Province, China.

**Keywords:** apelin, diabetic peripheral neuropathy, nerve conduction velocity, treatment, tumor necrosis factor-α

## Abstract

**Background:**

Diabetic peripheral neuropathy (DPN) is one of the most common chronic complications of diabetes. As apelin is an adipocytokine closely associated with diabetes, this study explored the clinical significance of serum apelin levels in patients with type 2 DPN before and after treatment.

**Methods:**

In total, 44 patients with T2DM without DPN (non-DPN group), 41 patients with DPN who received antihyperglycemic treatment (DPN-A group), 44 patients with DPN who received antihyperglycemic treatment combined with nutritional neurotherapy (DPN-B group), and 40 healthy control individuals (NC group) were selected continuously enrolled in the present study. Enzyme-linked immunosorbent assays (ELISA) were performed to determine serum levels of apelin and tumor necrosis factor-α (TNF-α). Related apelin, fasting blood glucose (FBG), glycosylated hemoglobin A1c, TNF-α, body mass index, fasting C peptide, and nerve conduction velocity (NCV) were recorded in each group before and after treatment.

**Results:**

Serum levels of apelin and TNF-α were higher in patients with diabetes than those in the NC group, as well as in the DPN group as compared to the non-DPN group; furthermore, some NCV values were significantly reduced in the DPN group. After treatment, the serum levels of apelin, TNF-α, and FBG reduced in patients with diabetes; moreover, apelin levels were found significantly lower in the DPN-B group as compared to the DPN-A group, while some NCV values significantly increased in the DPN-B group. Apelin was negatively correlated with part of NCV values and positively correlated with TNF-α and FBG (*P* < .01).

**Conclusion:**

Our results show that the increase in serum apelin levels is an important clinical reference index for DPN, while a decrease indicates that the DPN treatment is effective.

## 1 Introduction

Diabetic peripheral neuropathy (DPN) is the most neglected complication that can occur in different developmental stages of diabetes. Early DPN predominantly manifests with symptoms of sensory nerve involvement, including limb numbness, pain, and paresthesia, and gradually involves motor and autonomic nerves. This can further cause a weakening and even disappearance of the pain and temperature sensations, as well as a loss of vibratory sensation and tendon reflex function in patients.^[1]^ Approximately 10% to 20% of patients with type 2 diabetes present with diabetic neuropathy at the initial diagnosis and more than 50% of DPN patients demonstrate no typical clinical symptoms. A previous study has shown that approximately three-quarters of elderly patients are unaware of the presence of DPN.^[2]^ Another study has reported that painless and painful DPN were mainly undiagnosed in 81.9% and 57.0% of the subjects with T2DM, respectively.^[3]^ Owing to the insidious onset, and complex etiology, diagnosis of DPN is frequently delayed. DPN is derived not only from hyperglycemia, but can also be attributed to aging, hyperlipidemia, hypertension, and obesity. DPN is easily ignored or misdiagnosed, resulting in foot ulcers, gangrene of the lower limbs, and even amputation, highlighting the severe morbidity, mortality, and significant cost associated with this condition.^[4–7]^ Therefore, it is particularly important to accurately predict the diagnosis and development of DPN at earlier stages.

Apelin, a bioactive peptide extracted from the bovine stomach in 1998, is mainly involved in various physiological processes occurring in multiple organs.^[8–11]^ Apelin has been widely described as a beneficial adipocytokine with antidiabetic properties. Several studies have shown that apelin can improve insulin resistance (IR), inhibit arterial atherosclerosis (AS), and improve peripheral vascular perfusion and vascular function in the diabetic foot.^[12–17]^ Increasing evidence has also confirmed that apelin is a promising new therapeutic target in diabetes.^[18]^ Furthermore, apelin is widely present in neuronal cell bodies and fibers throughout the nerve axis.^[15]^ However, studies investigating apelin and peripheral neuropathy are limited. Therefore, in the present study, we aimed to explore the relationship between apelin and DPN before and after treatment, which could result in the development of new therapeutic targets.

## 2 Methods

### 2.1 Participants

This research was approved by the Ethics Committee of the Fifth People's Hospital of Jinan, with informed consent obtained from all participants. In total, 85 patients with type 2 diabetes mellitus (T2DM) and DPN (DPN group) who were admitted to the Department of Endocrinology of Jinan Fifth People's Hospital (Jinan, China), 44 T2DM subjects without DPN (non-DPN group), and 40 healthy subjects in the control group (NC group) were continuously selected from March 2018 to May 2019. The DPN group was composed of 44 males and 41 females, with an average age of 56.17 ± 6.28 years, and the mean duration of disease was 7.34 ± 1.99 years. The non-DPN group was composed of 24 males and 20 females, with an average age of 54.07 ± 7.76 years, and the mean duration of the disease was 5.33 ± 1.79 years. The healthy control group was composed of 22 males and 18 females, all of who were selected for study participation at the examination center; their mean age was 53.98 ± 5.50 years. There were was no statistical significance in the sex and age among groups (*P* > .05). The treatment was discontinued when severe adverse reactions appeared during the study.

### 2.2 Selection criteria

The selection criteria were in line with the 1999 World Health Organization diagnostic criteria for T2DM. The inclusion criteria were as follows:

1. Fasting blood glucose (FBG) of 8 to 12.0 mmol/L, with glycosylated hemoglobin A1c (HbA1c) ranging between 7% and 10%;
2. Autoantibodies associated with diabetes, including islet cell antibodies (ICAs), insulin autoantibodies (IAAs), and glutamate decarboxylase antibodies (GADAs) were all negative;
3. The diagnostic criteria for DPN included changes in clinical symptoms and the results of the electromyography;^[19,20]^

### 2.3 Exclusion criteria

Subjects who met one of the following conditions were excluded:

1. Patients with renal failure or abnormal liver, cardiac, or lung functions. The criteria for liver and kidney failure are that the values of transaminase and creatinine levels that exceeded the normal upper limit by 2.5 times; abnormal cardiac function was defined as brain natriuretic peptide (BNP) values that exceeded the normal upper limit by 2.5 times; normal lung function was defined as the absence of ventilatory disorders;
2. Pregnant women or breast-feeding patients;
3. Patients with type 1 diabetes, malignant tumors, cerebral infarction, or Guillain-Barre syndrome;
4. Patients who presented with long-term heavy drinking or drug exposure, which may cause peripheral nerve damage. Long-term heavy drinking is defined as an average daily alcohol intake greater than or equal to 40 g for men and greater than or equal to 20 g for women, for 5 consecutive years.^[21]^

### 2.4 Treatment methods

All patients received an insulin pump (S20153001, Medtronic, Minnesota, USA) for continuous hypodermic insulin aspart (No. 2017104592, Novo Nordisk PharmaCo., Ltd., Beijing, China) to reduce blood glucose. Each group reduced blood pressure and regulated blood lipid levels through regular exercise and diet management. Patients with hyperglycemia were treated with insulin therapy, while those with hypertension and dyslipidemia received antihypertensive and lipid-lowering treatment to adjust the FBG to ≤7.0 mmol/L, the postprandial 2 hours plasma glucose (2hPG) to ≤10.0 mmol/L, blood pressure (BP) to ≤140/90 mm Hg, total plasma cholesterol (TC) to <4.5 mmol/L, low-density lipoprotein cholesterol (LDL-c) to <2.6 mmol/L, and triglycerides (Tg) to <1.7 mmol/L. To avoid the influence of blood lipids and BP on study results, patients were required to achieve the therapeutic goal of blood lipids and BP within the normal range before enrolment. A randomization procedure was used to select the gender of male or female. We collected subjects with an age range between 40 and 75 years old, with no statistical differences in age and sex observed between the NC, non-DPN and DPN groups.

The DPN group was divided into the DPN-A group and the DPN-B group by employing the random number table method. The DPN-A group was treated with antihyperglycemic treatment. The DPN-B group received antihyperglycemic treatment and nutritional neurotherapy, composed of 0.5 mg intramuscular methylcobalamin (Sinopec H20058993, Nanjing Hailing Pharmaceutical Co., Ltd., Jiangsu, China) and intravenous lipoic acid (Sinopec H20066706, Jiangsu Shenlong Co., Ltd., Jiangsu, China) in 250 mL of sodium chloride, administered separately, once daily for 2 weeks. No serious adverse events were observed. Only 2 patients dropped out of the DPN-B group; 1 patient experienced an allergic reaction to lipoic acid, and another presented with pneumonia while under treatment.

### 2.5 Observational indexes

Height and weight were recorded and body mass index (BMI) was calculated. An automatic biochemical analyzer (Siemens, Munich, Germany) was used to detect FBG and blood lipids; HbA1c was determined by high-performance liquid chromatography using an HA-8160 automatic glycated hemoglobin analyzer (ARKRAY, Kyoto, Japan); Fasting C peptide (FCP) and diabetic antibodies (GADAs, ICAs, IAAs) were measured with a chemiluminescence method (Shenzhen New Industry Biomedical Engineering Co., Ltd., Guangdong, China). The serum of the subjects was collected and stored in a refrigerator (−70°C) for testing. Serum levels of apelin and TNF-α were detected by ELISA (Wuhan Huamei Biological Engineering Co., Ltd., Hubei, China). The symptoms and physical examination of the patients with DPN before and after treatment were evaluated.

### 2.6 Electromyography

All patients underwent Keypoint electromyography before and 1 day after treatment (model 9033A07, Dantec Company, Copenhagen, Denmark). Any source of interference should be avoided, and a quiet environment should be maintained during recording. The room temperature was controlled at 18°C to −25°C, and the skin temperature was maintained between 28°C and −32°C. The sensory conduction velocity (SCV) of the sural and ulnar nerve, and motor conduction velocity (MCV) of the ulnar nerve and common peroneal nerves were measured. The normal value of nerve conduction velocity (NCV) is based on the data provided by the reference standard.^[22]^ If the value exceeds 2.5 standard deviations of the normal value, it is judged as abnormal. DPN is diagnosed when 2 or more abnormal NCV values are recorded.

### 2.7 Statistical analyses

Experimental data were analyzed using Statistical Package for the Social Sciences (SPSS) 22.0 (IBM Corp., Armonk, NY). Quantitative data were expressed as mean ± standard deviation, and sample comparisons between the 3 groups were performed using one-way ANOVA or Kruskal–Wallis test, and Bonferroni correction was used. Student *t* test was used to analyze the sample mean. We used paired sample T before and after treatment. The relationships between continuous quantitative variables were determined by Pearson correlation analysis. *P* < .05 was considered statistically significant.

## 3 Results

### 3.1 Comparison of general data of 3 groups

No significant differences were observed in the general data, including age, sex, BP, serum creatinine, and blood lipids, among groups (*P* > .05; Table 1)

**Table 1:** Comparison of observation indexes of each group before treatment.

	NC (n = 40) (mean ± SD)	non-DPN(n = 44) (mean ± SD)	DPN (n = 83) (mean ± SD)	Value	Value
Age (yrs)	53.98 ± 5.50	54.07 ± 7.76	56.17 ± 6.28	2.252	.108
Gender (Male/Female)	22/18	24/20	44/41	0.697	.552
Course of disease (years)	/	5.33 ± 1.79	7.34 ± 1.99^Δ^	31.19	<.001
SBP (mm Hg)	127.98 ± 7.84	128.36 ± 9.17	129.82 ± 8.78	0.768	.465
DBP (mm Hg)	75.18 ± 6.64	76.43 ± 6.38	75.92 ± 7.99	0.314	.731
TC (mmol/L)	3.81 ± 1.50	3.65 ± 1.71	3.62 ± 1.61	1.447	.238
LDL-C (mmol/L)	2.05 ± 0.61	2.11 ± 0.53	2.16 ± 0.51	0.661	.573
TG (mmol/L)	1.20 ± 0.25	1.23 ± 0.23	1.29 ± 0.27	1.886	.155
sCr (umol/l)	71.93 ± 14.77	71.59 ± 15.53	71.88 ± 13.11	0.007	.993
BMI (kg/m^2^)	22.64 ± 1.99	24.33 ± 2.47^#^	24.68 ± 2.52^#^	10.198	<.001
FBG (mmol/)	5.41 ± 0.79	8.46 ± 1.47^#^	8.88 ± 1.64^#^	7.453	.001
2hPG (mmol/)	7.13 ± 1.28	11.41 ± 1.86^#^	11.64 ± 2.12^#^	8.097	<.001
FCP (ng/ml)	2.40 ± 0.41	2.31 ± 0.49	2.14 ± 0.44^#Δ^	5.195	.006
HbA1c (%)	5.17 ± 0.90	8.03 ± 1.04^#^	8.17 ± 1.16^#^	7.542	.001
apelin (ng/l)	348.36 ± 40.34	379.40 ± 38.34^#^	404.08 ± 50.87^#Δ^	20.617	<.001
TNF-α (pg/l)	10.13 ± 3.56	12.83 ± 2.96^#^	15.23 ± 3.89^#Δ^	27.847	<.001

### 3.2 Comparison of the levels of BMI, serum FBG, 2hPG, FCP, HbA1C, apelin, and tumor necrosis factor-α (TNF-α) levels before treatment

The BMI and serum levels of FBG, 2hPG, HbA1C, apelin, and TNF-α in the DPN and non-DPN groups were significantly higher than those determined in the NC group (*P* < .05); however, the difference between the DPN and non-DPN groups was not statistically significant (*P* > .05; Table 1), except for apelin and TNF-α. Serum levels of apelin and TNF-α were significantly higher in the DPN group than in the non-DPN group. The FCP level was significantly lower in the DPN group than in the non-DPN and NC groups (*P* < .05; Table 1).

### 3.3 Comparison of serum levels of FBG, FCP, apelin, and TNF-α before and after treatment among the 3 groups

The serum levels of FBG, apelin, and TNF-α in the non-DPN, DPN-A, and DPN-B groups were lower after treatment than before treatment (*P* < .05). After treatment, the serum apelin level in the DPN-B group decreased more significantly (*P* < .01) and was significantly lower than that of the DPN-A group (*P* < .05). In the DPN-B group, the serum FCP level was significantly higher after treatment than that before treatment (*P* < .05; Table 2).

**Table 2:** Comparison of FBG, FCP, Apelin and TNF-α before and after treatment.

groups	non-DPN (n = 44) (mean ± SD)	DPN-A (n = 41) (mean ± SD)	DPN-B (n = 42) (mean ± SD)	*F* value	*P* value
FBG (mmol/L)					
Before	8.46 ± 1.47	8.85 ± 1.63	8.91 ± 1.67	1.036	.358
After	6.46 ± 1.35#	6.54 ± 0.7^#^	6.49 ± 0.91^#^	0.462	.631
FCP (ng/mL)					
Before	2.31 ± 0.49	2.11 ± 0.48^∗^	2.18 ± 0.39^∗^	3.829	.043
After	2.35 ± 0.41	2.12 ± 0.34	2.32 ± 0.33^#Δ^	13.459	< .001
Apelin (ng/L)					
before	379.40 ± 38.34	400.88 ± 52.59^∗^	407.21 ± 49.57^∗^	4.413	.018
after	364.98 ± 35.03#	389.18 ± 47.71^#^	376.63 ± 43.52^#Δ^	3.481	.34
TNF-α (pg/L)					
Before	12.83 ± 2.96	14.56 ± 3.57^∗^	15.87 ± 4.12^∗^	7.872	.001
After	9.96 ± 2.11^#^	12.10 ± 2.49^#^	12.66 ± 3.52^#^	27.348	< .001

### 3.4 Comparison of nerve conduction velocity before and after treatment in DPN-A group, DPN-B group and non-DPN group

After antihyperglycemic treatment along with neurotherapy, the MCV of the common peroneal nerve and SCV of sural and ulnar nerves were significantly increased in the DPN-B group, and were significantly higher than those observed in the DPN-A group (*P* < .05); however, no significant differences were observed before and after treatment in the non-DPN group (*P* > .05). After treatment, both the MCV of the common peroneal nerve and SCV of the sural nerve were significantly increased in the DPN-A group (*P* < .05; Table 3), with no significant differences observed in the MCV and SCV of the ulnar nerve (*P* > .05; Table 3).

**Table 3:** Comparison of nerve conduction velocity before and after treatment.

groups	non-DPN (n = 44) (mean ± SD)	DPN-A (n = 41) (mean ± SD)	DPN-B (n = 42) (mean ± SD)	*F*-value	*P* value
Common peroneal nerve MCV (m/s)					
Before	49.91 ± 2.35	40.72 ± 2.96^∗^	40.62 ± 2.01^∗^	273.237	<.001
After	50.03 ± 2.15	42.32 ± 2.84^#^	44.74 ± 1.67^#Δ^	131.951	<.001
Ulnar nerve MCV (m/s)					
Before	49.32 ± 2.85	43.29 ± 3.41^∗^	42.93 ± 2.21^∗^	88.059	<.001
After	49.91 ± 2.93	42.72 ± 3.32	42.99 ± 2.24	152.823	<.001
Sural nerve SCV (m/s)					
Before	48.90 ± 2.29	41.44 ± 2.44^∗^	40.67 ± 2.38^∗^	158.888	<.001
After	49.27 ± 1.83	42.07 ± 1.93^#^	43.45 ± 2.19^#Δ^	138.976	<.001
Ulnar nerve SCV (m/s)					
Before	49.73 ± 2.01	42.47 ± 3.96^∗^	42.49 ± 2.93^∗^	107.12	<.001
After	49.91 ± 2.03	42.18 ± 3.63	45.67 ± 2.01^#Δ^	126.493	<.001

### 3.5 Correlation analysis

Apelin was positively correlated with FBG and TNF-α levels before and after treatment (*P* < .05; Table 4). A negative correlation was observed between apelin and the MCV of the common peroneal nerve and the SCV of the sural nerve (*P* < .05; Table 5).

**Table 4:** Correlation between apelin and FBG, HbA1c, BMI, TNF-α, FCP before and after treatment.

	BMI		TNF-α		FBG		HbA1c		FCP	
Before	r	p	r	p	r	p	r	p	r	p
Apelin treatment	0.069	.442	0.181	.041	0.185	.040	0.016	.861	0.018	.838
After treatment	–	–	0.191	.030	0.180	.043	–	–	0.045	.607

**Table 5:** Correlation between apelin and nerve conduction velocity (m/s) before and after treatment.

		CP-N		U-N		S-N		U-N	
		MCV (m/s)				SCV (m/s)			
Apelin		*r*	*P*	*r*	*P*	*r*	*P*	*r*	*P*
	Before treatment	−0.222	.012	−0.162	.070	−0.254	.004	−0.126	.160
	After treatment	−0.264	.003	−0.167	.065	−0.301	.001	−0.132	.105

## 4 Discussion

The mechanism underlying DPN could be related to hyperglycemia, accumulation of glycosylated compounds, activation of protein kinase C activity, enhancement of the polyol pathway activity, and enhancement of the amino hexose pathway activity. Metabolic pathways play a significant role in the development of DPN.^[23]^ And hyperglycemia is the main cause of DPN progression and mediates oxidative stress-induced cell damage, which is a unifying factor underlying mechanisms involved in DPN.^[4]^ Currently, clinical symptoms and electromyography have been used for diagnosis, and there exist no precise indicators for evaluating DPN. Therefore, risk prediction factors are a current hot spot and a key target of ongoing research.

Apelin has recently been identified as a polypeptide hormone reportedly linked to IR.^[13]^ A previous study showed elevated apelin levels in patients with T2DM, but it did not refer to the patients with DPN.^[24]^ Our study shows that the fasting plasma apelin level of patients with DPN is increased, while the correlation analysis shows that the apelin level are significantly correlated with FBG and TNF-α levels.

In this study, we have identified increased serum apelin levels in DPN patients as compared to healthy controls and non-DPN groups. This is consistent with a recently published comparative study evaluating patients with and without DPN.^[25]^ High apelin levels associated with hyperglycemia in DPN may be due to the key role of apelin in improving insulin sensitivity^[13]^ and increasing glucose use.^[12]^ In addition, a correlation between apelin, endothelial dysfunction and microangiopathy in subjects with diabetes has been suggested. In fact, a study by Martin et al, hinted that the common pathophysiology underlying the nervous system effects of DPN may be associated with microangiopathy.^[26]^ Furthermore, there is growing evidence that apelin could eliminate reactive oxygen species (ROS).^[15,27–31]^ An excess in ROS production is related to hyperglycemia, leading to oxidative stress on DPN, which could increase apelin levels, thus preventing further deterioration of DPN.

Otherwise, we have also found that the serum apelin levels decreased significantly after antihyperglycemic treatment along with nutritional neurotherapy than antihyperglycemic therapy. First, the increased apelin levels were reversed when blood glucose control was improved and glucotoxicity was relieved in patients with T2DM.^[32,33]^ Second, apelin expression decreased further with antioxidant treatment by lipoic acid and methylcobalamin which led to a reduction in the oxidative stress, and hence, decreasing the damage to the tissue cells. Lipoic acid is a powerful antioxidant with a unique disulfide bond, which can inhibit lipid peroxidation, eliminate oxygen free radicals, and reduce damage to tissue cells. Moreover, lipoic acid has reported beneficial effects in the treatment of diabetes complications.^[34]^ Additionally, methylcobalamin could promote myelinogenesis and axon regeneration through nucleic acid and protein synthesis, repairing peripheral nerve injury.^[35,36]^ Our study showed that the serum apelin level was closely associated with DPN and its expression was decreased after lipoic acid and methylcobalamin, thus preventing the progression of neuropathy. However, the exact mechanisms underlying these changes are ambiguous and needs to be further explored.

Apelin could play an important role in DPN due to its neuroprotective effects.^[15,27]^ Our study found that elevated apelin levels in T2DM subjects are negatively correlated with the MCV and SCV of multiple nerves (mainly lower-limb nerves), which is in accordance with 1 study that has reported that decreased NCV of the upper and lower limbs in patients with DPN could be improved by combining antihyperglycemic and neurotrophic treatments.^[5]^ Moreover, Zeng et al showed that apelin-13 could have a neuroprotective effect owing to the reduced ROS production induced by serum deprivation, mitochondrial depolarization, cytochrome C release, and activation of the apoptosis marker caspase-3.^[28]^ It has been suggested that apelin-13 inhibits the activation of caspase-3 and enhances the expression of Bcl-2 after stroke, suggesting that it has an antiapoptosis mechanism.^[27–29]^

On the other hand, inflammation seems to be involved in the peripheral nerve injury of DPN. In agreement with the results of numerous studies^[37–39]^ our study shows that serum TNF-α concentrations are higher in T2DM subjects with DPN than in healthy controls. These results confirm the catalytic role of TNF-α in the pathogenesis of DPN. TNF-α could up-regulate the expression of apelin mRNA in adipose tissue and its further increase concentration in plasma.^[40]^ This result is ensured by our study, suggesting that the serum apelin levels are positively correlated with TNF-α. Our report demonstrates the close internal relationship between apelin and inflammatory biomarkers, so the neuroprotective effect of apelin as inhibitor of inflammation needs further study. The unblinded treatment strategy is an open-label trial, which could easily induce bias. However, during the trial process, 2 independent individuals undertook the responsibilities of trial administrator and trial effect evaluator in an attempt to minimize bias. As the number of patients included in our study is relatively small, further multicenter studies with larger sample sizes are warranted to confirm these results.

## 5 Conclusion

As a summary, the findings of the present study suggest that apelin is closely related to T2DM and plays a specific role in the appearance and development of DPN. Moreover, an increase in apelin levels in subjects with DPN was found, that decreased after treatment to control glucose and nourish the nervous system. Apelin could have a protective effect on peripheral nerves and may be used as a risk indicator for the early detection of DPN in T2DM subjects, as well as to evaluate the effect of antihyperglycemic and neurotrophic treatments on T2DM subjects with DPN.

## Author contributions

**Conceptualization:** Shumiao Zhao, Shitao Wang, Hua Xu, Qi Wang.

**Data curation:** Hua Xu.

**Investigation:** Qi Wang, Xue Liu.

**Methodology:** Xuan Qiang Che, Xue Liu.

**Resources:** Xuan Qiang Che.

**Software:** Qian Wang.

**Supervision:** Qian Wang, Shumiao Zhao.

**Writing – original draft:** Hua Xu, Qi Wang.

**Writing – review & editing:** Shumiao Zhao, shitao wang.

## References

[1] Pop-Busui R Boulton AJ Feldman EL. Diabetic neuropathy: a position statement by the american diabetes association. Diabetes Care 2017;40:136–54. 27999003 10.2337/dc16-2042 PMC6977405

[2] Bongaerts BW Rathmann W Heier M. Older subjects with diabetes and prediabetes are frequently unaware of having distal sensorimotor polyneuropathy: the KORA F4 study. Diabetes Care 2013;36:1141–6. 23275355 10.2337/dc12-0744 PMC3631873

[3] Ziegler D Landgraf R Lobmann R. Painful and painless neuropathies are distinct and largely undiagnosed entities in subjects participating in an educational initiative (PROTECTstudy). Diabetes Res Clin Pract 2018;139:147–54. 29518491 10.1016/j.diabres.2018.02.043

[4] Downs CA Faulkner MS. Toxic stress, inflammation and symptomatology of chronic complications in diabetes. World J Diabetes 2015;6:554–65. 25987953 10.4239/wjd.v6.i4.554 PMC4434076

[5] Kim BBJ Kim S. Adaptation of perturbation to postural control in individuals with diabetic peripheral neuropathy. Int J Occup Saf Ergon 2018;01–6. 10.1080/10803548.2018.1494771 29996729

[6] Dixit S Gular K Asiri F. Effect of diverse physical rehabilitative interventions on static postural control in diabetic peripheral neuropathy: a systematic review. Physiother Theory Pract 2018;01–12. 10.1080/09593985.2018.1491078 29979897

[7] Zhang X Fang C Li X. Clinical characteristics and risk factors of diabetic peripheral neuropathy of type 1 diabetes mellitus patients. Diabetes Res Clin Pract 2017;129:97–104. 28521198 10.1016/j.diabres.2017.04.016

[8] Liu HT Chen M Yu J. Serum apelin level predicts the major adverse cardiac events in patients with ST elevation myocardial infarction receiving percutaneous coronary intervention. Medicine (Baltimore) 2015;94:e449. 25634182 10.1097/MD.0000000000000449 PMC4602953

[9] Wang YZ Fan J Zhong B. Apelin: a novel prognostic predictor for atrial fibrillation recurrence after pulmonary vein isolation. Medicine (Baltimore) 2018;97:e12580. 30278567 10.1097/MD.0000000000012580 PMC6181607

[10] Kasai A Shintani N Oda M. Apelin is a novel angiogenic factor in retinal endothelial cells. Biochem Biophys Res Commun 2004;325:395–400. 15530405 10.1016/j.bbrc.2004.10.042

[11] Tatemoto K Hosoya M Habata Y. Isolation and characterization of a novel endogenous peptide ligand for the human APJ receptor. Biochem Biophys Res Commun 1998;251:471–6. 9792798 10.1006/bbrc.1998.9489

[12] Dray C Knauf C Daviaud D. Apelin stimulates glucose utilization in normal and obese insulin-resistant mice. Cell Metab 2008;8:437–45. 19046574 10.1016/j.cmet.2008.10.003

[13] Yue P Jin H Aillaud M. Apelin is necessary for the maintenance of insulin sensitivity. Am J Physiol Endocrinol Metab 2010;298:E59–67. 19861585 10.1152/ajpendo.00385.2009 PMC2806109

[14] Sato T Suzuki T Watanabe H. Apelin is a positive regulator of ACE2 in failing hearts. J Clin Invest 2013;123:5203–11. 24177423 10.1172/JCI69608 PMC3859384

[15] Chen D Lee J Gu X. Intranasa delivery of apelin-13 is neuroprotective and promotes angiogenesis after ischemic stroke in mice. ASN Neuro 2015;7:01–15. 10.1177/1759091415605114 PMC4580122 26391329

[16] Samura M Morikage N Suehiro K. Combinatorial treatment with apelin-13 enhances the therapeutic efficacy of a preconditioned cell-based therapy for peripheral ischemia. Sci Rep 2016;6:19379. 26763337 10.1038/srep19379 PMC4725909

[17] McKenzie JA Fruttiger M Abraham S. Apelin is required for non-neovascular remodeling in the retina. Am J Pathol 2012;180:399–409. 22067912 10.1016/j.ajpath.2011.09.035 PMC3244602

[18] Zhu S Sun F Li W. Apelin stimulates glucose uptake through the PI3K/Akt pathway and improves insulin resistance in 3T3-L1 adipocytes. Mol Cell Biochem 2011;353:305–13. 21461612 10.1007/s11010-011-0799-0

[19] Callaghan BC Price RS Chen KS. The importance of rare subtypes in diagnosis and treatment of peripheral neuropathy: a review. JAMA Neurol 2015;72:1510–8. 26437251 10.1001/jamaneurol.2015.2347 PMC5087804

[20] Shabeeb D Najafi M Hasanzadeh G. Electrophysiological measurements of diabetic peripheral neuropathy: a systematic review. Diabetes Metab Syndr 2018;12:591–600. 29610062 10.1016/j.dsx.2018.03.026

[21] Philippe Mathurin P Hadengue A Bataller R. EASL clinical practical guidelines: management of alcoholic liver disease. J Hepatol 2012;57:399–420. 22633836 10.1016/j.jhep.2012.04.004

[22] Tang XF. Clinical Electrophysiology of the Nervous System. Vol 97. 2002;Beijing: People's Military Medical Publishing House, 84–91. (In Chinese).

[23] Tang HY Jiang AJ Ma JL. Understanding the signaling pathways related to the mechanism and treatment of diabetic peripheral neuropathy. Endocrinology 2019;160:2119–27. 31318414 10.1210/en.2019-00311

[24] Li L Yang G Li Q. Changes and relations of circulating visfatin, apelin, and resistin levels in normal, impaired glucose tolerance, and type 2 diabetic subjects. Exp Clin Endocrinol Diabetes 2006;114:544–8. 17177135 10.1055/s-2006-948309

[25] Bilir B Ekiz Bilir B Yilmaz I. Association of apelin, endoglin and endocan with diabetic peripheral neuropathy in type 2 diabetic patients. Eur Rev Med Pharmacol Sci 2016;20:892–8. 27010147

[26] Martin CL Albers JW Pop-Busui R. Neuropathy and related findings in the diabetes control and complications trial/epidemiology of diabetes interventions and complications study. Diabetes Care 2014;37:31–8. 24356595 10.2337/dc13-2114 PMC3868000

[27] Zeng XJ Yu SP Zhang L. Neuroprotective effect of the endogenous neural peptide apelin in cultured mouse cortical neurons. Exp Cell Res 2010;316:1773–83. 20152832 10.1016/j.yexcr.2010.02.005 PMC3155990

[28] Tang SY Xie H Yuan LQ. Apelin stimulates proliferation and suppresses apoptosis of mouse osteoblastic cell line MC3T3-E1 via JNK and PI3-K/Akt signaling pathways. Peptides 2007;28:708–18. 17109997 10.1016/j.peptides.2006.10.005

[29] Yang Y Zhang X Cui H. Apelin-13 protects the brain against ischemia/reperfusion injury through activating PI3K/Akt and ERK1/2 signaling pathways. Neurosci Lett 2014;568:44–9. 24686182 10.1016/j.neulet.2014.03.037

[30] Gu Q Zhai L Feng X. Apelin-36, a potent peptide, protects against ischemic brain injury by activating the PI3K/Akt pathway. Neurochem Int 2013;63:535–40. 24083989 10.1016/j.neuint.2013.09.017

[31] Qiu J Wang X Wu F. Low dose of apelin-36 attenuates ER stress-associated apoptosis in rats with ischemic stroke. Front Neurol 2017;8:556. 29085332 10.3389/fneur.2017.00556 PMC5650706

[32] Tang C Koulajian K Schuiki I. Glucose-induced beta cell dysfunction in vivo in rats: link between oxidative stress and endoplasmic reticulum stress. Diabetologia 2012;55:1366–79. 22396011 10.1007/s00125-012-2474-8

[33] Jonas JC Bensellam M Duprez J. Glucose regulation of islet stress responses and beta-cell failure in type 2 diabetes. Diabetes Obes Metab 2009;11: Suppl 4: 65–81. 19817790 10.1111/j.1463-1326.2009.01112.x

[34] Bartkoski S Day M. Alpha-lipoic acid for treatment of diabetic peripheral neuropathy. Am Fam Physician 2016;93:786. 27175957

[35] Kuwabara S Nakazawa R Azuma N. Intravenous methylcobalamin treatment for uremic and diabetic neuropathy in chronic hemodialysis patients. Intern Med 1999;38:472–5. 10411351 10.2169/internalmedicine.38.472

[36] Jiang DQ Li MX Wang Y. Effects of prostaglandin E1 plus methylcobalamin alone and in combination with lipoic acid on nerve conduction velocity in patients with diabetic peripheral neuropathy: a meta-analysis. Neurosci Lett 2015;594:23–9. 25800109 10.1016/j.neulet.2015.03.037

[37] Ristikj-Stomnaroska D Risteska-Nejashmikj V Papazova M. Role of inflammation in the pathogenesis of diabetic peripheral neuropathy. Open Access Maced J Med Sci 2019;7:2267–70. 31592273 10.3889/oamjms.2019.646 PMC6765096

[38] Duksal T Tiftikcioglu BI Bilgin S. Role of inflammation in sensory neuropathy in prediabetes or diabetes. Acta Neurol Scand 2016;133:384–90. 26346888 10.1111/ane.12474

[39] Calle MC Fernandez ML. Inflammation and type 2 diabetes. Diabetes Metab 2012;38:183–91. 22252015 10.1016/j.diabet.2011.11.006

[40] Daviaud D Boucher J Gesta S. TNFalpha up-regulates apelin expression in human and mouse adipose tissue. Faseb j 2006;20:1528–30. 16723381 10.1096/fj.05-5243fje

